# Super-wide viewing-zone holographic 3D display using a convex parabolic mirror

**DOI:** 10.1038/s41598-018-29798-5

**Published:** 2018-07-27

**Authors:** Yusuke Sando, Kazuo Satoh, Takahiro Kitagawa, Makoto Kawamura, Daisuke Barada, Toyohiko Yatagai

**Affiliations:** 1 Utsunomiya University, Center for Optical Research & Education, Utsunomiya, 321-8585 Japan; 2 Utsunomiya University, Graduate School of Engineering, Utsunomiya, 321-8585 Japan; 3 Osaka Research Institute of Industrial Science and Technology, Izumi center, Izumi, 594-1157 Japan

## Abstract

To enlarge both horizontal (azimuthal) and vertical (zenithal) viewing zones simultaneously, a convex parabolic mirror is placed after passing through the hologram. Viewers perceive a three-dimensional (3D) object inside the parabolic mirror as a virtual image by capturing the wavefront radially reflected from the parabolic mirror. The optical experiment using the convex parabolic mirror has demonstrated an extremely wide viewing zone with an azimuthal range of 180° and zenithal range of 90°. The viewing zone and the shape of the parabolic surface are analyzed. The hologram is designed considering the parabolic mirror reflection, and its diffraction calculation method based on Fermat’s principle is also proposed.

## Introduction

A floating display of a three-dimensional (3D) object in midair has attracted the attention of many researchers because of its potential application in fields such as virtual reality and augmented reality^1,2^. A holographic 3D display provides realistic images with all the physiological perception factors: there is neither 3D sickness, nor the necessity of wearing customized glasses^3,4^. Moreover, the holographic 3D display realizes the natural and continuous regeneration of motion parallax. Motion parallax yields much higher realistic sensation than only the binocular parallax, on which commercially available 3D displays are based. Sufficient motion parallax would be indispensable for realizing various types of applications, such as simulation training, virtual 3D printing, and virtual 3D museums, which require a free view-point observation different from 3D televisions or movies.

**Figure 1:**
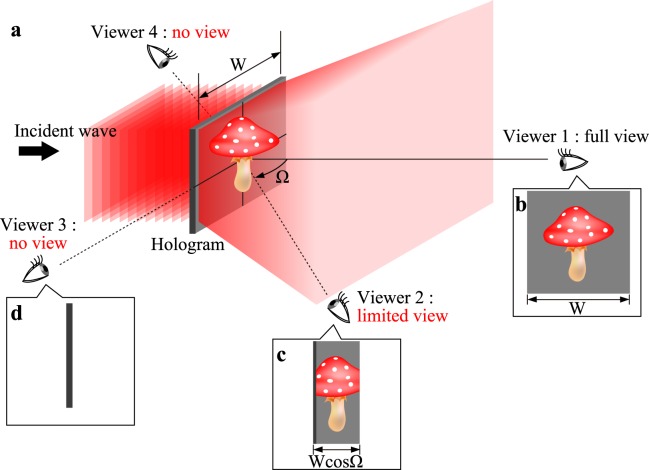
Schematic of the fundamental limitation in planar holograms. (**a**) shows a perspective view in the reconstruction. (**b**–**d**) are individual fields of view from various observation directions.

To exert the effect of motion parallax fully, a wide viewing zone is inevitable. The viewing zone is an area within which viewers can observe 3D images. However, the viewing zone is strictly limited by the shape of holograms in principle, even though a sufficient diffraction angle is realized. For example, in the planar hologram, which is the most common in holography, the viewing zone is confined to the forward region of the hologram, as shown in Fig. 1(a). It is never possible to observe 3D images from either the sides or the opposite direction of the hologram. Even more unfortunately, the reconstructible size of 3D images or field of view decreases cosinoidally with the angle Ω between the normal of the hologram and the observation direction. Figure 1(b–d) schematically illustrates this defect. The reconstructible size depends on the observation positions. These are the fundamental limitations of planar holograms.

**Figure 2:**
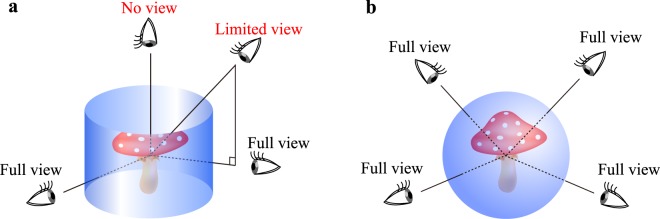
Reconstruction from non-planar holograms: (**a**) cylindrical hologram and (**b**) spherical hologram.

The breakthrough of this problem came in the 1960s. Jeong proposed cylindrical holography, where the hologram is not planar but cylindrical^5^. Because of the cylindrical shape, multiple viewers can properly observe the 3D images simultaneously from all horizontal directions^6–8^. Since a horizontally full motion parallax is reproduced, the reconstructible size in the horizontal direction does not depend on the observation direction. A multiplex hologram, which is a holographic stereogram recorded on a cylindrical surface, is a type of cylindrical hologram^9^. Its medical application has also been reported^10^. However, in cylindrical holography, the vertical property is the same as that of the planar hologram: neither the vertical viewing zone nor vertical reconstructible size is improved as shown in Fig. 2(a). From the perspective of the shape, a spherical hologram is considered to have an ultimate shape because it can yield the perfect viewing zone, as shown in Fig. 2(b). The reconstructible size is constant without any dependence on the observation directions. However, it is extremely difficult to realize spherical holograms. In addition to the fabrication of spherical holograms, the generation of reconstructing spherical waves with large divergent angles is a considerably difficult task. Although some researchers proposed spherical holograms for the perfect viewing zone, they just showed fast calculation algorithms for diffraction onto the spherical surfaces, and no experimental results were provided^11–13^. Moreover, to utilize the holographic 3D display practically, a 3D animation or a movie of 3D objects is essentially required. To show such a 3D movie, the hologram pattern has to be dynamically modulated by displaying the hologram on an electrically accessible spatial light modulator (SLM)^14,15^. The SLM can display not only optically recorded digital holograms but also computer-generated holograms (CGHs) synthesized by simulating the optical diffraction in a computer^16–18^. However, all SLMs are planar. To the best of our knowledge, no spherical SLM is commercially available at present.

This paper presents a tactical method enabling a viewing zone wider than that of a hemispherical hologram, in spite of the planar hologram used. A key point for that is the introduction of a convex parabolic mirror inserted between a hologram and viewers. The viewers can perceive a 3D object as a virtual image near the focal point of the parabolic mirror by capturing the wavefronts after the parabolic mirror reflection. Because the convex parabolic mirror reflects the incident wave radially, this method enables a drastic enlargement of the viewing zone in both horizontal and vertical directions. Our proposal can yield a quasi-perfect viewing zone in spite of the planar hologram used.

## Results

### Spherical wave generation with a convex parabolic mirror

**Figure 3:**
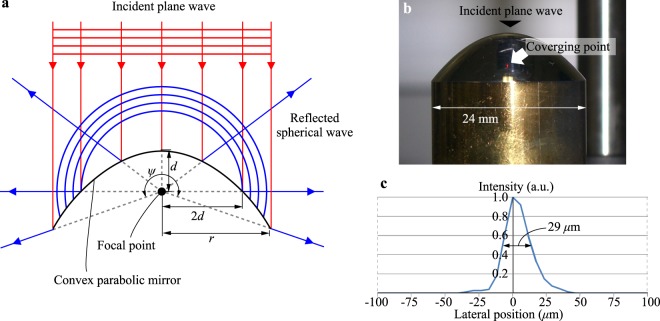
Spherical wave generation with a large divergent angle. (**a**) shows the geometrical conversion of a plane wave into a spherical wave by the parabolic mirror reflection. (**b**) is the picture of the convex parabolic mirror illuminated by the plane wave. The picture was captured under room lighting to clarify the shape of the parabolic mirror and the virtually converging point. (**c**) is a graph showing the horizontal lateral profile of the virtually converging point.

The viewing zone enlargement in our proposal is based on a convex parabolic mirror reflection. As shown in Fig. 3(a), in the convex parabolic mirror, there is a geometrical property that an incident plane wave is converted into a spherical wave diverging from a focal point of the parabolic mirror after the mirror reflection. The divergent angle of the reflected spherical wave is determined by the ratio of the radius *r* to the focal length *d* of the convex parabolic mirror. The greater the ratio *r*/*d* is set, the larger the divergent angle becomes. When *r* is greater than 2*d*, the divergent angle exceeds 180°, which is unrealizable with a spherical wave generation based on refraction using an objective lens with high numerical aperture. The horizontal (azimuthal) range of the reflected spherical wave is 360° owing to rotational symmetry of the parabolic mirror.

A result of the spherical wave generation by a convex parabolic mirror is shown in Fig. 3(b). This convex parabolic surface, whose material was brass, was fabricated by machining and polishing. A thin aluminum layer was deposited by using electron beam evaporation to enhance the reflectivity. The radius and the focal length of the convex parabolic mirror are 12 mm and 5 mm, respectively. In Fig. 3(b), a plane wave, which is invisible, enters the convex parabolic mirror vertically downward. A digital camera captures the spherical wave generated by the parabolic mirror reflection. Because the focus of the camera is set to the focal point of the convex parabolic mirror, a very small but sparkling point is imaged, as shown in Fig. 3(b). This smallness and brightness indicates proper spherical wave generation. The full width at half maximum of the horizontal lateral profile of this sparkling point is 29 *μ*m, as is shown in Fig. 3(c).

### Holographic 3D display system with a perfect viewing zone

**Figure 4:**
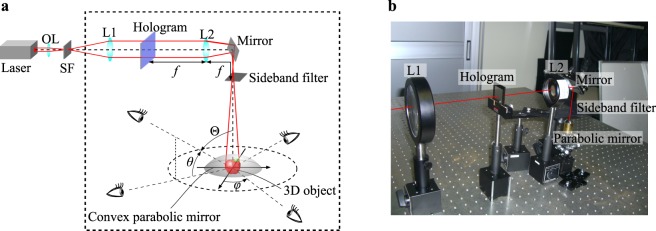
Holographic 3D display system for an extremely wide viewing zone. (**a**) is a schematic of an optical system with a convex parabolic mirror. OL: objective lens, SF: spatial filter, L1: lens, and L2: lens. (**b**) is the picture of the practical optical system. The dashed square part of (**a**) is photographed.

To apply the above geometrical property of a convex parabolic mirror to the enlargement of the viewing zone, an optical setup, as shown in Fig. 4(a), is proposed. A plane wave, which is generated by an objective lens, a spatial filter, and a lens L1, illuminates a hologram. The wavefront modulated by the hologram is Fourier-transformed by the lens L2, and half of it is filtered by a sideband filter on the Fourier plane to remove the 0th-order diffraction wave and the conjugate wave. Then, the wavefront enters the convex parabolic mirror vertically downward, and diverges radially by the parabolic mirror reflection. Viewers capture the radially reflected wavefront and observe the 3D object inside the parabolic mirror. The 3D object is reconstructed as a virtual image near the focal point of the parabolic mirror. When the observation direction is identified by an azimuthal angle *φ* and an elevation angle *θ*, as shown in Fig. 4(a), the horizontal (azimuthal) viewing zone becomes 180°, which is the range of the angle *φ*. On the other hand, the vertical viewing zone, which is the range of the zenithal angle Θ = 90° − *θ*, is determined by the ratio between the radius and the focal length of the parabolic mirror. A hologram used in this method is a Fourier transform hologram, and the hologram pattern needs to be calculated and designed properly by considering the convex parabolic mirror reflection.

### Optically reconstructed images

**Figure 5:**
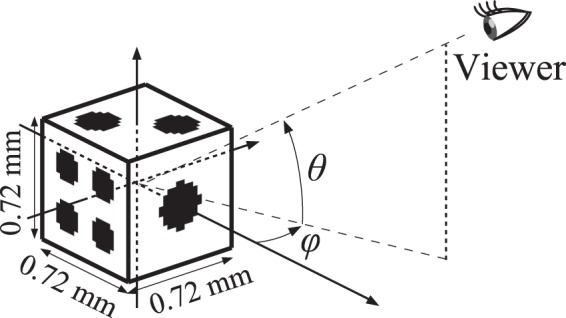
Schematic of the 3D object used and the coordinate system for the observation.

**Figure 6:**
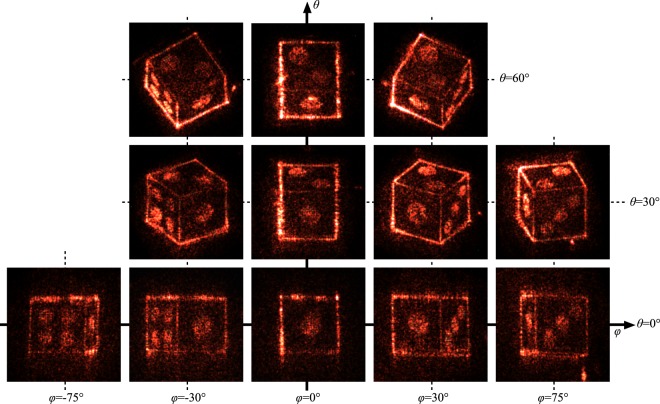
Optically reconstructed images captured from various observation directions indicated by *θ* and *φ*.

An optical experiment with quasi-perfect viewing zone was demonstrated. The picture of the practical optical system is shown in Fig. 4(b). The 3D object used for this experiment is a die whose sides are 0.72 mm in length, as shown in Fig. 5, and which consists of 684 point light sources. The focal length of lens L2 is 150 mm. The radius and the focal length of the parabolic mirror are 12 mm and 5 mm, respectively. This yields a vertical viewing zone of more than 90°. The hologram used for this experiment was of the binary amplitude-modulation type, and was fabricated by photolithography and wet etching. The pixel number and the pixel pitch of the hologram were 8,192 × 8,192 and 4 *μ*m × 4 *μ*m, respectively. Under these conditions, the holographic 3D display was constructed. The reconstructed images were captured by a digital camera from various observation directions, as shown in Fig. 6. In Fig. 6, the horizontal and vertical positions represent the azimuthal angle *φ* and elevation angle *θ* of the capturing camera, respectively. The disparity images are reconstructed appropriately according to the camera position (a movie showing motion parallax is provided as a supplementary video online). The observable range in the azimuthal and elevation directions are 180° and 90°, respectively. This viewing zone is extremely large compared with other preceding viewing-zone enlargement methods^19–21^. Some reconstructed images in the directions of *θ* = 90° (zenith), *φ* = ±90°, and so on, could not be captured because of the physical contact of optical elements and the camera, although they were surely reconstructed properly. Moreover, occlusion culling or hidden surface removal^22–24^ functioned properly. For instance, the three pips of the die do not show up in the reconstructed images captured from the directions where *φ* is less than 0, although the three pips are located on the face opposite to the four pips in the 3D object data.

## Discussion

This paper presents a method to realize quasi-perfect viewing zone in holographic 3D display. Our method can achieve a wider viewing zone than that of a hemispherical hologram. The horizontal (azimuthal) and vertical (zenithal) ranges of the viewing zone are, in principle, 360° and more than 90°, respectively. However, in the optical demonstration, the azimuthal range was just 180°. The reason for this difference is that half of the wavefront was spatially filtered by a sideband filter, as shown in Fig. 4(a). This sideband filter was inserted to remove unnecessary waves such as the 0th-order diffraction wave and the conjugate wave. In other words, if a grayscale phase-type hologram were used instead of the amplitude-type hologram, such unnecessary waves would never emerge and the sideband filter would not be required. Because it is technically possible to use the phase-type hologram, our method is capable of realizing a perfect azimuthal viewing zone of 360°. In addition, a grayscale phase-type hologram enhances the quality of the reconstructed images.

The size of the reconstructed image is also an important factor. In general, there is a trade-off relation between the viewing zone and the reconstructible size in a holographic 3D display. Our method focuses on enlarging the viewing zone at the expense of the size of the reconstructed image. The size of the die used for the optical demonstration was just 0.72 mm in length, as is shown in Fig. 5. It is difficult to observe the reconstructed image without magnification. In order to enlarge the reconstructible size with keeping the quasi-perfect viewing zone, the pixel pitch of a hologram must be reduced, or the focal length of the convex parabolic mirror must be increased with keeping the ratio among the radius, focal length, and size of the hologram constant. These requirements are necessary to avoid an aliasing error while sampling the wavefront on the hologram plane. Eventually, both the approaches offer the same problem — increase in the pixel number of the hologram. To increase the pixel number, some methods such as spatial multiplexing methods^25–28^, time division methods^20,29–31^, and a combination of these methods^32^ have been proposed. A drastic increase in the substantive pixel number is already technically possible, and these pixel-number increasing techniques can be applied to our method because the hologram used in our proposed method is planar. Therefore, our approach achieves the quasi-perfect viewing zone and the reconstruction of large 3D objects simultaneously, which cannot be accomplished through the conventional approaches.

In our method, a 3D object is reconstructed inside the convex parabolic mirror as a virtual image because our method requires reflection on the parabolic mirror. In addition to the reflector, the parabolic mirror functions as an aperture for field stop. The reconstructible size of the 3D object is limited by this aperture whose effective shape depends on the observation direction: the field of view depends on the zenithal angle Θ of the observation direction. For example, assuming that the radius *r* of the parabolic mirror is twice that of the focal length *d*, the aperture is a circle of radius 2*d* in the case of Θ = 0°. The aperture deforms with an increase in Θ. When Θ is 90°, the aperture forms a parabolic half-moon with height *d*. This dependency on the observation direction is not desirable. This dependency in our method is, however, much superior to that in a conventional planar hologram. As is shown in Fig. 1, the effective aperture or the field of view of the conventional planar hologram diminishes cosinoidally, and it disappears completely for the observation from the side of the hologram. There is another point to note with respect to reflection on the parabolic mirror. Owing to the reflection on the parabolic mirror, viewers can observe the 3D object from an extremely large viewing zone, but they can never touch the 3D object. Before touching the 3D object, their hands will hit the parabolic mirror. This problem can be solved by using a concave parabolic mirror instead of a convex parabolic mirror. The concave parabolic mirror also converts an incident plane wave into a spherical wave; however, the spherical wave does not diverge but converges. Its focal point is located in midair. If the concave parabolic mirror is used, the 3D object will be reconstructed near the focal point as a real image. Thus, a floating 3D display in midair can be realized, although either the horizontal (azimuthal) viewing zone is limited to less than 180° by using a half-cut concave parabolic mirror, or the vertical (zenithal) viewing zone is limited to less than 90° to prevent the concave parabolic mirror from blocking the reflected wavefront.

## Methods

### Design of the convex parabolic mirror

**Figure 7:**
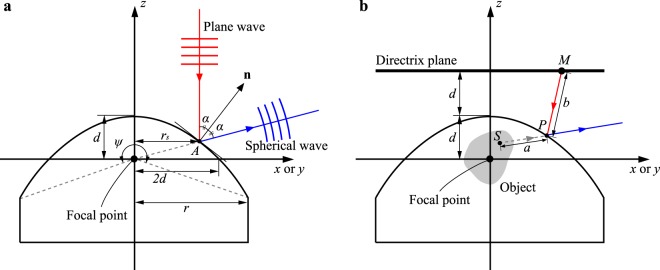
Diagrams of the (**a**) divergent angle of the generated spherical wave and (**b**) optical path considering the parabolic mirror reflection.

The size and focal length of a convex parabolic mirror determines the divergent angle of a spherical wave after reflection. As shown in Fig. 7(a), it is assumed that the focal point of the parabolic mirror is located at the origin of the coordinate system (*x*, *y*, *z*), and the z-axis is set as the rotation axis of the parabolic mirror. In this configuration, the convex parabolic surface whose focal length is *d* is expressed as

1 $$z=-\,\frac{{x}^{2}+{y}^{2}}{4d}+d.$$

The light ray entering the parabolic surface vertically downward at the point *A*(*x*_*s*_, *y*_*s*_, *z*_*s*_) proceeds in the other direction after the parabolic mirror reflection. The normal vector **n** of the parabolic surface at the point *A* is given by **n** = (*x*_*s*_, *y*_*s*_, 2*d*), and the angle between the incident direction (z axis) and the normal vector **n** is *α*(*r*_*s*_) = tan^−1^ (*r*_*s*_/2*d*), where ${r}_{s}=\sqrt{{x}_{s}^{2}+{y}_{s}^{2}}$ is the distance between the point *A* and the z axis. Since the law of reflection should be satisfied at the reflection at point *A*, the reflected light ray propagates in the direction of 2*α* from the z-axis. Thus, when the radius of the parabolic mirror is denoted by *r*, the divergent angle *ψ* is represented by

2 $$\psi =4\alpha (r)=4\,{\tan }^{-1}(\frac{r}{2d}).$$

Given that the radius *r* is more than 2*d*, the resulting divergent angle exceeds 180°, which corresponds to the fact that the zenithal range of the viewing zone exceeds 90°.

### Calculation method of a hologram considering the parabolic mirror reflection

Our method requires a hologram pattern designed by calculating inverse diffraction from a 3D object to the hologram plane considering the reflection on the convex parabolic mirror. Inverse diffraction calculation is implemented based on the geometrical optics to take such reflection into account. A 3D object is assumed to be an aggregation of point light sources. Here, the optical path from one point light source *S* to one point *M* on the directrix plane of the parabolic surface via the parabolic surface is examined as is shown in Fig. 7(b). Note that since the 3D object is to be reconstructed inside the parabolic mirror as a virtual image, the traveling direction of the light ray from point *S* to the parabolic surface is forward, whereas the direction from the parabolic surface to the point *M* is backward. To obtain the wavefront at point *M* contributed from point light source *S*, the optical path length is indispensable, which means that the reflection point *P* on the parabolic surface has to be identified. When the coordinates of the points *S*, *P*, and *M* are represented by (*x*_*S*_, *y*_*S*_, *z*_*S*_), (*x*_*P*_, *y*_*P*_, *z*_*P*_), and (*x*_*M*_, *y*_*M*_, *z*_*M*_), respectively, the optical path length *l* is expressed as follows:

3 $$l({x}_{P},{y}_{P},{z}_{P})=a-b,$$

where *a* and *b* represents distances from point *S* to point *P* and from point *P* to point *M*, respectively, and they are given by

4 $$a=\sqrt{{({x}_{P}-{x}_{S})}^{2}+{({y}_{P}-{y}_{S})}^{2}+{({z}_{P}-{z}_{S})}^{2}}$$

5 $$b=\sqrt{{({x}_{M}-{x}_{P})}^{2}+{({y}_{M}-{y}_{P})}^{2}+{({z}_{M}-{z}_{P})}^{2}}.$$

According to Fermat’s principle, the light ray follows the shortest optical path^33^. Therefore, the coordinate (*x*_*P*_, *y*_*P*_, *z*_*P*_) of the reflection point *P* is given as the solution which minimizes *l*(*x*_*P*_, *y*_*P*_, *z*_*P*_) subject to $$g({x}_{P},{y}_{P},{z}_{P})\,=$$$${z}_{P}+({x}_{P}^{2}+{y}_{P}^{2})\mathrm{/4}d-d=0$$. This minimization problem with the constraint can be solved by a method of Lagrange multiplier^34^. The Lagrange function $$ {\mathcal L} ({x}_{P},{y}_{P},{z}_{P},\lambda )$$ is defined by

6 $$ {\mathcal L} ({x}_{P},{y}_{P},{z}_{P},\lambda )=l({x}_{P},{y}_{P},{z}_{P})-\lambda g({x}_{P},{y}_{P},{z}_{P}),$$

where *λ* is a Lagrange multiplier. If the point *P*(*x*_*P*_, *y*_*P*_, *z*_*P*_) minimizes *l*, the following equations are to be satisfied:

7 $$\frac{\partial  {\mathcal L} }{\partial {x}_{P}}=\frac{{x}_{P}-{x}_{S}}{a}+\frac{{x}_{M}-{x}_{P}}{b}-\frac{\lambda {x}_{P}}{2d}=0$$

8 $$\frac{\partial  {\mathcal L} }{\partial {y}_{P}}=\frac{{y}_{P}-{y}_{S}}{a}+\frac{{y}_{M}-{y}_{P}}{b}-\frac{\lambda {y}_{P}}{2d}=0$$

9 $$\frac{\partial  {\mathcal L} }{\partial {z}_{P}}=\frac{{z}_{P}-{z}_{S}}{a}+\frac{{z}_{M}-{z}_{P}}{b}-\lambda =0$$

10 $$\frac{\partial  {\mathcal L} }{\partial \lambda }=-\,g({x}_{P},{y}_{P},{z}_{P})=-\,{z}_{P}-\frac{{x}_{P}^{2}+{y}_{P}^{2}}{4d}+d=0.$$

As *z*_*P*_ and *λ* can be rewritten as functions of two variables *x*_*P*_ and *y*_*P*_ using Eqs (9) and (10), the above equations are practically non-linear simultaneous equations with two variables (*x*_*P*_, *y*_*P*_). Because these nonlinear simultaneous equations cannot be solved analytically, Newton’s method for two variables was used to solve the simultaneous equations numerically^35^. From the numerical solution of (*x*_*P*_, *y*_*P*_, *z*_*P*_), the optical path length *l* and the wavefront at point *M*, contributed from the point light source *S*, can be easily calculated. By repeating this procedure for all point light sources constituting the 3D object and all the sampling points on the directrix plane, the wavefront *h* on the directrix plane is obtained. Since the hologram used in this study is a Fourier-transforming-type hologram, the half part of the wavefront *h* is filtered and is then inversely Fourier-transformed by fast Fourier transform. Finally, the hologram pattern is obtained as its binarized real part.

Occlusion culling is important to reconstruct complicated and realistic 3D objects. Our method can incorporate occlusion culling very easily by simply selecting the light rays that are not occluded by other point light sources. Light rays behind the point light sources are discarded during wavefront calculation.

### Data availability

The data that support the findings of this study are available from the corresponding author upon request.

## Electronic supplementary material

Motion parallax
